# A Brief Description of Medical Education Master Program in Shiraz University of Medical Sciences

**Published:** 2015-10

**Authors:** FARNAZ TAKMIL, MITRA AMINI, PARISA NABEIEI, LEILA BAZRAFKAN, MOHAMMAD REZA DEHGHANI, RITA REZAEE, SHIRIN GHANAVATI, JAVAD KOJURI

**Affiliations:** Quality Improvement in Clinical Education Research Center, Shiraz University of Medical Sciences, Shiraz, Iran

As Medical sciences become more advanced and grow each day, so do the challenges of medical education. To overcome the new obstacles which are an inseparable part of science evolution, it is necessary to renew and improve the educational system .Achieving this, requires individuals who are educated in the new methods of learning and therefore can lead others through educational system development. During the past decade, these facts have led many medical universities toward establishing and pursuing new master and PhD. programs of medical education. As a Pioneer in the field of medical education and the first teacher training center in EMRO since 1972, Shiraz University of Medical Sciences (SUMS) has also contributed to educational system transformation by establishing master courses in medical education since 2008, with a goal of not only updating faculties` educational knowledge but also training efficient educational experts. In SUMS, these courses which are also mentioned in Dr. ARA Tekian’s articlein Chicago University (1), were designed based on reviewing what Some of the greatest universities in the field of education such as Dundee, Maastricht and Chicago University have done. The courses last for five semesters and are available for all medical and paramedical faculty members, GPs and paramedical masters. To enter the program applicants must send their resume including their degree, previous education, and published articles. Moreover, they must also take part in an entrance exam which evaluates their knowledge of general English language along with their educational knowledge and computer skills. These courses are conducted using the newest methods and references of learning such as “A practical guide for medical teachers” (2). The students are also asked to study the most recent AMME Guidelines which are provided for them. According to students’ work field, in each semester, proper homework has been considered to improve what they’ve been instructed in practice.

After becoming educational experts, these students can be hired in different sections of education departments, acting as educational ambassadors, initiating educational leaders & curriculums designers which, in turn, can little by little change the culture and face of education, hoping that these changes may someday result in spurring medical educational system. 
